# Eigenvector centrality dynamics are related to Alzheimer’s disease pathological changes in non-demented individuals

**DOI:** 10.1093/braincomms/fcad088

**Published:** 2023-03-28

**Authors:** Luigi Lorenzini, Silvia Ingala, Lyduine E Collij, Viktor Wottschel, Sven Haller, Kaj Blennow, Giovanni Frisoni, Gaël Chételat, Pierre Payoux, Pablo Lage-Martinez, Michael Ewers, Adam Waldman, Joanna Wardlaw, Craig Ritchie, Juan Domingo Gispert, Henk J M M Mutsaerts, Pieter Jelle Visser, Philip Scheltens, Betty Tijms, Frederik Barkhof, Alle Meije Wink

**Affiliations:** Department of Radiology and Nuclear Medicine, Amsterdam University Medical Centre, Vrije Universiteit, Amsterdam Neuroscience, Amsterdam 1081 HV, The Netherlands; Amsterdam Neuroscience, Brain Imaging, Amsterdam, The Netherlands; Department of Radiology and Nuclear Medicine, Amsterdam University Medical Centre, Vrije Universiteit, Amsterdam Neuroscience, Amsterdam 1081 HV, The Netherlands; Amsterdam Neuroscience, Brain Imaging, Amsterdam, The Netherlands; Department of Radiology, Copenhagen University Hospital Rigshospitalet, 2100 Copenhagen, Denmark; Cerebriu A/S, Copenhagen 1127, Denmark; Department of Radiology and Nuclear Medicine, Amsterdam University Medical Centre, Vrije Universiteit, Amsterdam Neuroscience, Amsterdam 1081 HV, The Netherlands; Amsterdam Neuroscience, Brain Imaging, Amsterdam, The Netherlands; Department of Radiology and Nuclear Medicine, Amsterdam University Medical Centre, Vrije Universiteit, Amsterdam Neuroscience, Amsterdam 1081 HV, The Netherlands; Amsterdam Neuroscience, Brain Imaging, Amsterdam, The Netherlands; CIMC—Centre d’Imagerie Médicale de Cornavin, 1201Genève, Switzerland; Department of Surgical Sciences, Radiology, Uppsala University, Uppsala 751 85, Sweden; Department of Radiology, Beijing Tiantan Hospital, Capital Medical University, Beijing, 100070, P. R. China; Department of Psychiatry and Neurochemistry, Institute of Neuroscience and Physiology, the Sahlgrenska Academy at the University of Gothenburg, Mölndal 431 41, Sweden; Clinical Neurochemistry Laboratory, Sahlgrenska University Hospital, Mölndal 405 30, Sweden; Laboratory Alzheimer’s Neuroimaging & Epidemiology, IRCCS Istituto Centro San Giovanni di Dio Fatebenefratelli, Brescia 25125, Italy; University Hospitals and University of Geneva, Geneva 1205, Switzerland; Université de Normandie, Unicaen, Inserm, U1237, PhIND ‘Physiopathology and Imaging of Neurological Disorders’, Institut Blood-and-Brain @ Caen-Normandie, Cyceron, 14000 Caen, France; Department of Nuclear Medicine, Toulouse University Hospital, Toulouse 31300, France; ToNIC, Toulouse NeuroImaging Center, University of Toulouse, Inserm, UPS, Toulouse 31300, France; Centro de Investigación y Terapias Avanzadas, Neurología, CITA-Alzheimer Foundation, San Sebastián 20009, Spain; German Center for Neurodegenerative Diseases (DZNE), Munich 81377, Germany; Centre for Clinical Brain Sciences, The University of Edinburgh, Edinburgh, UK; Department of Medicine, Imperial College London, London, UK; Centre for Clinical Brain Sciences, The University of Edinburgh, Edinburgh, UK; UK Dementia Research Institute at Edinburgh, University of Edinburgh, Edinburgh, UK; Centre for Dementia Prevention, The University of Edinburgh, Scotland, UK; Scottish Brain Sciences, Edinburgh, UK; Barcelonaβeta Brain Research Center (BBRC), Pasqual Maragall Foundation, Barcelona, Spain; CIBER Bioingeniería, Biomateriales y Nanomedicina (CIBER-BBN), Madrid, Spain; IMIM (Hospital del Mar Medical Research Institute), BarcelonaSpain; Universitat Pompeu Fabra, Barcelona, Spain; Department of Radiology and Nuclear Medicine, Amsterdam University Medical Centre, Vrije Universiteit, Amsterdam Neuroscience, Amsterdam 1081 HV, The Netherlands; Amsterdam Neuroscience, Brain Imaging, Amsterdam, The Netherlands; Ghent Institute for Functional and Metabolic Imaging (GIfMI), Ghent University, Ghent, Belgium; Department of Neurology, Alzheimer Center Amsterdam, Amsterdam Neuroscience, Vrije Universiteit Amsterdam, Amsterdam UMC, Amsterdam, the Netherlands; Alzheimer Center Limburg, Department of Psychiatry & Neuropsychology, School of Mental Health and Neuroscience, Maastricht University, Maastricht, The Netherlands; Division of Neurogeriatrics, Department of Neurobiology, Care Sciences and Society, Karolinska Institutet, Stockholm, Sweden; Department of Neurology, Alzheimer Center Amsterdam, Amsterdam Neuroscience, Vrije Universiteit Amsterdam, Amsterdam UMC, Amsterdam, the Netherlands; Department of Neurology, Alzheimer Center Amsterdam, Amsterdam Neuroscience, Vrije Universiteit Amsterdam, Amsterdam UMC, Amsterdam, the Netherlands; Department of Radiology and Nuclear Medicine, Amsterdam University Medical Centre, Vrije Universiteit, Amsterdam Neuroscience, Amsterdam 1081 HV, The Netherlands; Institutes of Neurology and Healthcare Engineering, University College London, London, UK; Department of Radiology and Nuclear Medicine, Amsterdam University Medical Centre, Vrije Universiteit, Amsterdam Neuroscience, Amsterdam 1081 HV, The Netherlands

**Keywords:** functional connectivity, eigenvector centrality, amyloid, preclinical Alzheimer’s disease

## Abstract

Amyloid-β accumulation starts in highly connected brain regions and is associated with functional connectivity alterations in the early stages of Alzheimer’s disease. This regional vulnerability is related to the high neuronal activity and strong fluctuations typical of these regions. Recently, dynamic functional connectivity was introduced to investigate changes in functional network organization over time. High dynamic functional connectivity variations indicate increased regional flexibility to participate in multiple subnetworks, promoting functional integration. Currently, only a limited number of studies have explored the temporal dynamics of functional connectivity in the pre-dementia stages of Alzheimer’s disease. We study the associations between abnormal cerebrospinal fluid amyloid and both static and dynamic properties of functional hubs, using eigenvector centrality, and their relationship with cognitive performance, in 701 non-demented participants from the European Prevention of Alzheimer’s Dementia cohort. Voxel-wise eigenvector centrality was computed for the whole functional magnetic resonance imaging time series (static), and within a sliding window (dynamic). Differences in static eigenvector centrality between amyloid positive (A+) and negative (A-) participants and amyloid-tau groups were found in a general linear model. Dynamic eigenvector centrality standard deviation and range were compared between groups within clusters of significant static eigenvector centrality differences, and within 10 canonical resting-state networks. The effect of the interaction between amyloid status and cognitive performance on dynamic eigenvector centrality variability was also evaluated with linear models. Models were corrected for age, sex, and education level. Lower static centrality was found in A+ participants in posterior brain areas including a parietal and an occipital cluster; higher static centrality was found in a medio-frontal cluster. Lower eigenvector centrality variability (standard deviation) occurred in A+ participants in the frontal cluster. The default mode network and the dorsal visual networks of A+ participants had lower dynamic eigenvector centrality variability. Centrality variability in the default mode network and dorsal visual networks were associated with cognitive performance in the A- and A+ groups, with lower variability being observed in A+ participants with good cognitive scores. Our results support the role and timing of eigenvector centrality alterations in very early stages of Alzheimer’s disease and show that centrality variability over time adds relevant information on the dynamic patterns that cause static eigenvector centrality alterations. We propose that dynamic eigenvector centrality is an early biomarker of the interplay between early Alzheimer’s disease pathology and cognitive decline.

## Teaser

Lorenzini *et al*. demonstrate widespread dynamic functional connectivity impairments in relationship with Alzheimer’s disease pathological changes in non-demented individuals. This work suggests that initial amyloid deposition affects eigenvector centrality temporal patterns by reducing the involvement of functional hubs in different network dynamics, therefore reducing functional integration, and promoting cognitive deterioration.

## Graphical Abstract

**Graphical abstract:**
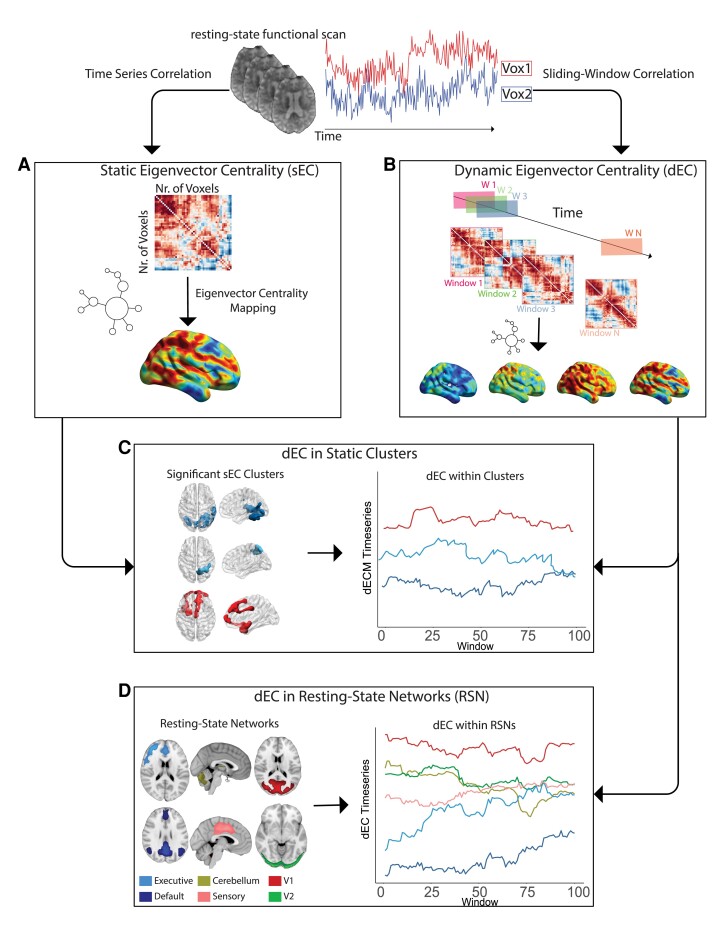

See Skouras (https://doi.org/10.1093/braincomms/fcad104) for a scientific commentary on this article.

## Introduction

Amyloid deposition is considered to be the first pathological event of Alzheimer’s disease (AD), followed by neurofibrillary tangles and neuronal injury.^1^ Previous work has shown that early amyloid burden, in pre-symptomatic stages of AD, occurs within specific cortical regions of the default mode network (DMN),^2^ causing decreased posterior and increased anterior DMN functional connectivity (FC),^3^ and leading to subsequent cognitive impairment. Emerging evidence suggests that this selective vulnerability to amyloid deposition is related to the high neuronal activation and, notably, to the high variability in the temporal pattern of neuronal fluctuations, typical of these regions.^4^ Dynamic connectivity has recently emerged from the observation that FC strongly fluctuates over time, and therefore assuming its stationarity might not entirely capture the complexity of this phenomenon.^5^ These short-time scale variations in functional network organization have been observed to be related to the alternation of intra- and inter-functional networks connectivity, suggesting canonical resting-state networks (RSNs) to be only transiently isolated from each other, and therefore promoting functional integration.^6^ However, the influence of amyloid deposition on FC and on its short time scale properties, i.e. dynamic patterns, remains unclear.

Graph analytic methods provide a framework to analyze topological properties of the functional connectome,^7^ by representing the brain as a set of nodes and edges, i.e. brain regions and (functional) connections, respectively.^8^ Eigenvector centrality (EC) is a hierarchical measure of the influence of a node in the network, computed as the sum of the centralities of a node’s neighbors, which allows the reliable identification of ‘hub’ regions in functional brain networks.^9,10^ Differences in voxel-wise EC have been reported in Alzheimer's patients.^9^ Those changes were related to cognitive impairment,^9^ abnormal amyloid levels, and CSF phosphorylated-tau (p-tau)–Aβ1–42 ratios in cognitively unimpaired individuals.^11,12^

Dynamic investigations of functional networks have revealed meaningful variations of graph properties over time. Increased time-related variation of dynamic graph properties, such as EC, suggest greater participation of brain regions in different subnetworks, thus promoting functional integration.^6,13^ However, relatively little has been done to elucidate how amyloid deposition alters FC dynamics in the early stages of AD, and whether this is reflected in cognitive performance. We hypothesize that amyloid deposition impairs both long- and short-time scales EC, here referred to as static and dynamic EC, and expect greater dynamic alterations in relationship to initial cognitive impairment.

We address the association of functional EC with cerebrospinal fluid (CSF) amyloid load and initial cognitive impairment.. To this end, we assessed both static and dynamic EC relation with CSF Aβ1–42 positivity, and its interaction with cognitive performance, in a large sample of non-demented participants from the European Prevention of Alzheimer Dementia (EPAD) cohort study.^14^

## Materials and methods

### Study participants

We used the v1500.0 baseline data release of EPAD cohort study.^14^ EPAD study general inclusion criteria are described in Solomon *et al*.^15^ and include: age above 50 years and no diagnosis of dementia [clinical dementia rating (CDR) < 1]. Using the demographic, cognitive, neuroimaging, fluid biomarkers of the study,^15^ we selected individuals who had CSF, cognitive evaluation, three-dimensional T1-weighted (3D T1w) and resting-state functional magnetic resonance imaging (rs-fMRI) data available, resulting in a sample size of *n* = 736. Institutional review boards of each participating center approved the EPAD study.

### CSF analysis

CSF biomarkers were quantified using a harmonized pre-analytical protocol. Analyses were performed with the fully automatised Roche cobas Elecsys System at the Clinical Neurochemistry Laboratory, Mölndal, Sweden.^15^ Concentrations of amyloid-β (Aβ1–42) were determined using the manufacturer’s guidelines.

### Amyloid status and amyloid-tau classification

Following previous works on the same cohort,^16^ CSF Aβ1–42 levels <1000 pg/mL were used to define amyloid positivity (A+). Further, CSF p-tau levels >27 pg/mL were used to define tau positivity (T+). Four amyloid-tau (AT) groups were derived to define A-T-, A+T-, A+T+, and A-T+ participants.

### Cognitive testing

The EPAD Neuropsychological Examination battery covers relevant cognitive domains; data were collected with standardized procedures on a tablet.^17,18^ Performed tests included the mini-mental state examination (MMSE),^19^ the CDR scale,^20^ and the Repeatable Battery for the Assessment of Neuropsychological Status (RBANS).^21^ The RBANS test covers five cognitive domains: attention, language, delayed memory, immediate memory, and visuo-constructional indices.^21^

### MRI acquisition and pre-processing

MRI acquisition and pre-processing details are given in Lorenzini *et al*.^22^ 3D T1w images were pre-processed using the structural module of ExploreASL.^23^ Lesion filling on 3D T1w images using the Lesion Segmentation Toolbox v2.0.15^24^ was followed by tissue segmentation with the computational anatomy toolbox (CAT) 12.^25^ For the rs-fMRI data (200 volumes, TR = 2 s, TE = 30 ms), magnetic field related inhomogeneity was estimated using a previously described method,^26^ implemented in FMRIB Software Library (FSL) as topup,^27^ to correct geometric distortion. Functional image preprocessing included motion correction, spatial smoothing (FWHM = 4 mm) and high-pass temporal filtering (100 s). Each subject’s rs-fMRI image was then registered to the individual 3D T1w scan and resampled to Montreal Neurological Institute standard space to a voxel dimension of 4 × 4 × 4 mm. Scans with a mean framewise displacement of more than 2 standard deviations (SDs) measured over time were excluded from further analysis, reducing the sample size to *n* = 701.

### Functional eigenvector centrality

EC evaluates nodes in the network stating that a node is important when it is linked to other important nodes.^28^ We computed voxel-wise EC within the gray matter (>0.2 as measured by the CAT12 gray matter probabilistic segmentation) for each rs-fMRI scan using the fast eigenvector centrality mapping (fastECM) toolbox (https://github.com/amwink/bias/tree/master/matlab/fastECM).^29^ EC computation requires the calculation of a voxel-wise connectivity matrix to obtain its eigenvector.^28^ The fastECM toolbox provides a fast and computationally efficient EC calculation by computing matrix-vector products, without having to compute or store the connectivity matrix. First, we computed voxel-wise EC over the whole time series—referred to as static EC. To assess temporal centrality dynamics, we computed voxel-wise EC in 100 partially-overlapping sliding windows of 100 time points each—referred to as dynamic EC. A lower limit for the window length to avoid the identification of spurious fluctuations has been identified as the longest wavelength in functional magnetic resonance imaging (fMRI) time courses;^30,31^ previous works have shown EC fluctuation differences in relation to age, sex, and clinical symptoms using similar window lengths to ours.^13,32^ EC maps were computed for each time window and concatenated to yield a four-dimensional (4D) dynamic centrality time series for each participant.

### Statistical analysis

All statistical analyses were performed using FSL^27^ and R (https://cran.r-project.org/), version 4.0.3. Chi-square test and *t*-test were initially used to compare demographics and clinical characteristics between A+ and A- participants and between AT groups.

#### Static eigenvector centrality differences between amyloid groups

To assess whether EC maps computed over the whole time series differed between amyloid groups, we compared static voxel-wise EC maps between amyloid positive (A+) and amyloid negative (A-) participants using general linear model (GLM) analyses with sex and age as covariates. A permutation-based method (implemented in FSL randomise) was used to compute cluster significance at *P* < 0.05, using threshold-free cluster enhancement to correct for spatial dependencies.^33,34^

#### Static eigenvector centrality differences between AT groups

Next, we investigated differences in static EC between AT groups. We excluded participants in the A-T+ group as considered suspected non-AD pathology. As a confirmation of the previous analysis, we compared A+ and A- groups again but on this subset of participants. Subsequently, GLM analyses were used to investigate differences between the three AT groups, with sex and age as covariates. A permutation-based method (implemented in FSL randomise) was used to compute cluster significance at *P* < 0.05, using threshold-free cluster enhancement to correct for spatial dependencies.^33,34^

#### Dynamic eigenvector centrality within static clusters

We assessed EC variability over time within the between-group differences in the static EC analysis. Three maps of significant clusters from the previous analysis were used to calculate average within-cluster dynamic EC time series from the 4D individuals’ dynamic EC files. From these time series, we assessed dynamic EC variability by computing (i) SD of EC values across time windows and (ii) minimum and maximum EC range across time windows. SD and range within the three clusters were compared between A+ and A- and between AT groups through linear models correcting for age and sex.

#### Dynamic eigenvector centrality in canonical resting-state networks

**Figure 1:**
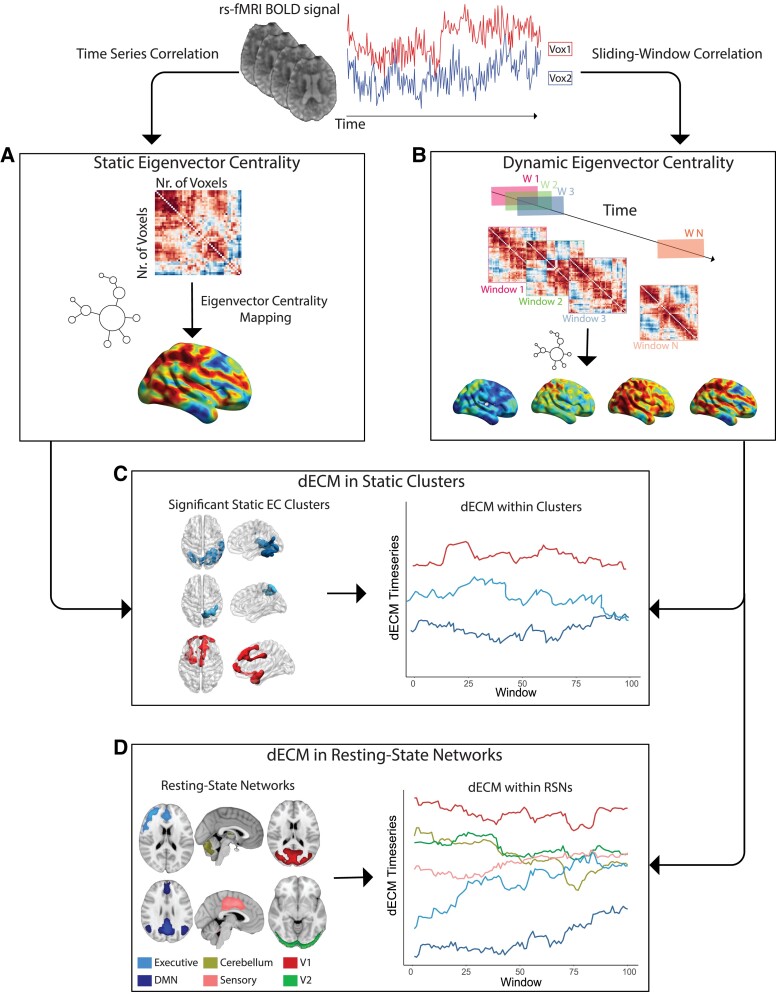
**Static and dynamic fastECM pipeline.** From the pre-processed rs-fMRI BOLD time series (upper row) static ECM is first computed at the voxel level on the whole time series voxel-by-voxel correlation matrix (**A**). Subsequently, ECM is performed within partially overlapping sliding windows used to segment the fMRI time series (**B**). Dynamic ECM time series are then extracted for the clusters showing statistical significant differences in the static EC analysis (**C**) and within resting-state networks (**D**). Abbreviations: rs-fMRI = resting-state functional magnetic resonance imaging; BOLD = blood oxygenation level dependent; ECM = eigenvector centrality mapping; dECM = dynamic ECM; ICA = independent component analysis; DMN = Default mode network; V1 = Visual network 1; V2 = Visual network 2.

To evaluate dynamic EC characteristics independently of static results, we computed dynamic EC time series within 10 canonical RSNs^35^ using a dual regression approach.^36^ First, subject-specific EC time courses of RSNs were obtained by spatial regression of the full set of RSN masks against each participant’s dynamic EC time series. The resulting time courses were then regressed onto the participants’ centrality time series to obtain subject-specific spatial maps. Temporal SD and range were computed for each RSN mask. The resulting dynamic EC RSN spatial maps were compared between amyloid positive (A+) and amyloid negative (A-) participants using GLM analyses with sex and age as covariates. A permutation-based method (implemented in FSL randomise) was used to compute cluster significance at *P* < 0.05, using threshold-free cluster enhancement to correct for spatial dependencies.^33,34^ For the networks showing spatial differences, we further evaluated whether temporal variability measures (SD and range) within those networks differed based on amyloid status and AT groups using GLM models corrected for age and sex. The used pipeline is shown in Fig. 1.

#### Relation of eigenvector centrality with cognitive performance

The relation between dynamic EC temporal variability, SD and range, and cognitive performance was then assessed. For each of the dynamic RSN showing significant differences between amyloid groups, we used linear regression models investigating the effect of MMSE and the five RBANS domains on temporal variability measures (SD and range). All models included an interaction term between the variable of interest and amyloid status and were corrected for age, sex, and years of education. Statistical significance was set at *P* < 0.05. The same analysis was repeated using the mean static EC within RSNs as the dependent variable.

#### Data availability

The data used in this manuscript are available upon request at https://ep-ad.org/open-access-data/access/

## Results

### Sample characteristics

In total, 701 participants were included in this study. Demographics, clinical, and neuropsychological characteristics of the sample are shown in Table 1. Mean age was 64.6 (SD = 7.01) and 409 participants (58.3%) were female. Mean MMSE score was 28.7 (SD = 1.53), and 15.7% participants had a CDR of 0.5. There were 232 (33.1%) participants who were classified as A+. Overall, worse cognitive performance was observed in the A+ group, with higher percentages of CDR = 0.5 participants, and lower score in the RBANS total scale, sum of index and attention index (Table 1). Participants characteristics based on AT status are reported in Supplementary Table 1.

**Table 1:** Demographic and clinical characteristics of amyloid positive and negative individuals

	Amyloid negative (A-)	Amyloid positive (A+)
	*n* = 469	*n* = 232
Males, *N* (%)	183 (39)	109 (47.0)
Age, Mean (SD)	64.34 (6.93)	65.23 (7.17)
CDR = 0.5, *N* (%)	59 (12.7)	50 (21.6)
MMSE, Mean (SD)	28.85 (1.44)	28.65 (1.69)
Hippocampal Volume in mm3, Mean (SD)	2926.59 (472.40)	2817.46 (490.26)
Years of Education, mean (SD)	14.69 (3.79)	14.57 (3.86)
RBANS		
Total Scale, Mean (SD)	104.98 (12.54)	102.27 (13.65)
Sum of Index, Mean (SD)	518.50 (43.16)	508.51 (48.99)
Attention, Mean (SD)	100.03 (15.28)	97.66 (15.71)
Delayed Memory, Mean (SD)	103.70 (12.53)	101.49 (15.09)
Language, Mean (SD)	99.66 (9.62)	98.12 (11.00)
Visuo-Construction, Mean (SD)	109.22 (15.26)	107.44 (15.07)
Immediate Memory, Mean (SD)	105.95 (12.99)	103.80 (14.71)

Maximum CDR in the EPAD cohort is 0.5, therefore the number (and percentage) of CDR = 0.5 is reported. Abbreviations: SD = Standard Deviation; MMSE = Mini-mental state examination; CDR = clinical dementia rating scale; RBANS = The Repeatable Battery for the Assessment of Neuropsychological Status.

### Comparing eigenvector centrality between amyloid and AT groups

**Figure 2:**
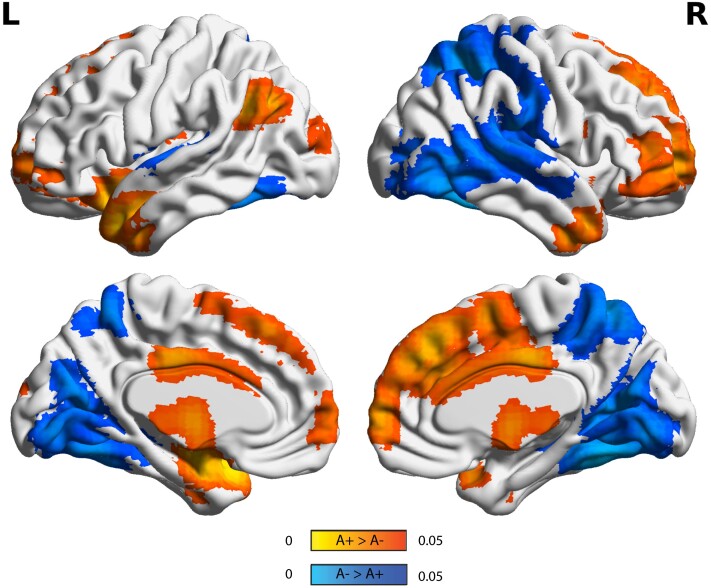
**Static eigenvector centrality differences in amyloid groups (A-T+ excluded).** Surface plots of *P*-values in statistical significant clusters. Orange shows regions where ECM A+ > A- and blue shows regions where ECM A- > A+. Upper one is the lateral view, lower row is the medial view, for the left hemisphere (left column) and right hemisphere (right column), respectively. Abbreviations: L = Left; R = Right; A+ = Amyloid positive; A- = Amyloid negative.

**Static EC between Amyloid groups.** Three clusters showed differences between the A+ and A- groups (*P* < 0.05) in the static EC analysis (Fig. 2). Lower centralities in A+ participants were observed for a parietal cluster covering the right precuneus and the posterior parietal lobule, and for an occipital cluster extending to the medial and ventral portions of the inferior and middle occipital lobe. A third cluster, covering mediofrontal regions and extending to left anterior-temporal areas, had higher EC values in A+ participants. Results from this same analysis when also including A-T+ did not show substantial changes and are reported in Supplementary Fig. 1. Raw mean voxel-wise eigenvector centrality mapping (ECM) per group is shown in Supplementary Fig. 2.

**Figure 3:**
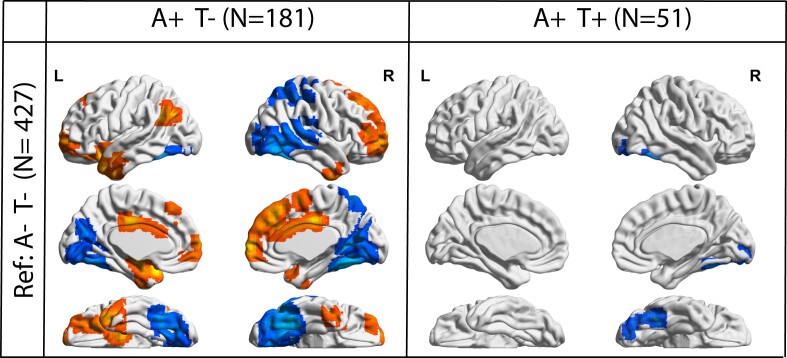
**Static eigenvector centrality differences in AT groups.** Surface plots of *P*-values in statistical significant clusters. Left: Regions showing increased (red) or decreased (blue) EC in A+T- participants compared to A-T-. Right: Regions showing increased (red) or decreased (blue) EC in A+T+ participants compared to A-T-. Upper one is the lateral view, middle row is the medial view, lower row is ventral view. Abbreviations: Ref = Reference group; A-T- = Amyloid negative Tau negative; A+T- = Amyloid positive Tau negative; A+T+ = Amyloid positive Tau positive.

**Static EC between AT groups.** When comparing voxel-wise EC between AT groups, A+T- participants showed a pattern of alteration similar to the one revealed by the previous analysis. Two posterior clusters, covering the parietal and occipito-temporal lobe, demonstrated reduced EC in A+T- compared to controls (A-T-). One anterior cluster, including frontal and anterior-temporal regions, showed increased EC. In the A+T+ groups, we found that one cluster in the ventral temporal lobe showed decreased EC compared to A-T-, while no increases of connectivity were found. No difference was found between A+T- and A+T+ groups (Fig. 3).

**Figure 4:**
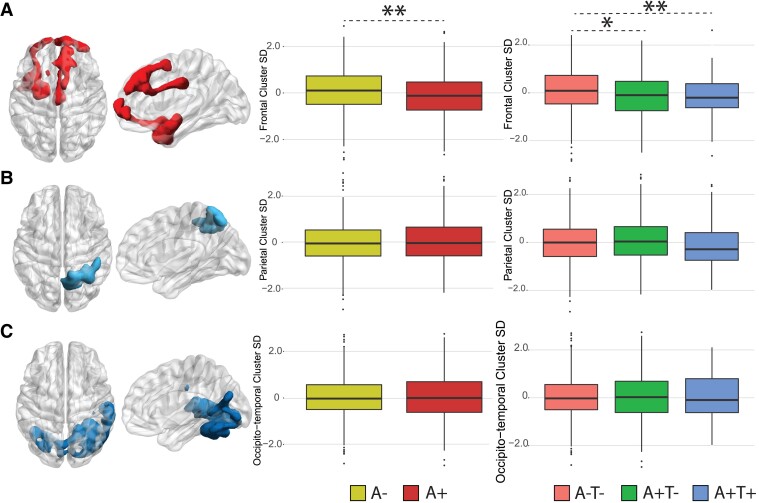
**Dynamic EC variability measured within static clusters.** Rendering of significant clusters from the static analysis and differences (linear models) in within-cluster dynamic EC standard deviation between amyloid for the frontotemporal (**A**), parietal (**B**), and occipito-temporal (**C**) clusters. Abbreviations: SD = Standard deviation; A+ = Amyloid positive; A- = Amyloid negative; A-T- = Amyloid negative Tau negative; A+T- = Amyloid positive Tau negative; A+T+ = Amyloid positive Tau positive. * = *P* < 0.05, ** = *P* < 0.01, *** = *P* < 0.001.

**Dynamic EC in Static Clusters.** A reduction of dynamic EC SD in A+ participants was observed in the frontotemporal cluster (*P* < 0.005**, β = -0.24). When looking at AT groups, the same cluster showed reduced SD in A+T- (*P* < 0.05*, β = -0.19) and A+T+ (*P* < 0.001**, β = -0.41) groups compared to controls (A-T-). No differences were found between A+T- and A+T+. EC temporal variability within the parietal and occipito-temporal clusters did not differ between amyloid groups **(**Fig. 4**)**.

**Figure 5:**
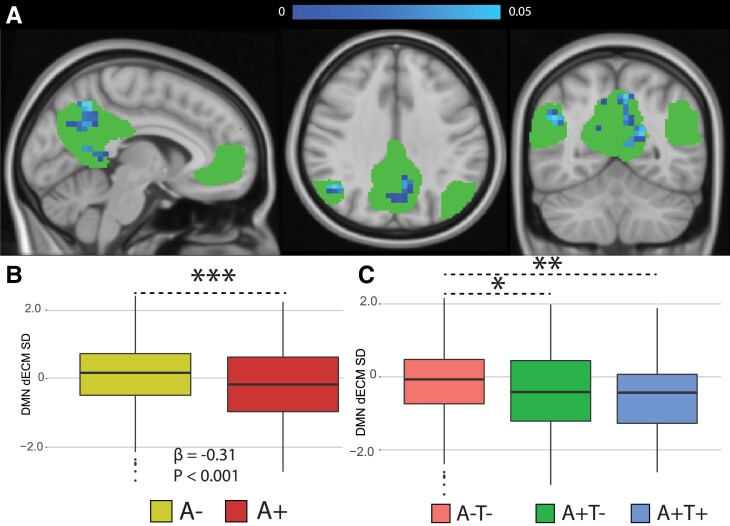
**Dynamic functional connectivity eigenvector centrality in the default mode network.** (**A**) Statistical significant differences showing lower dynamics in amyloid positive participants (in blue). Bottom-row: Differences in DMN dynamic EC standard deviation between amyloid groups (**B**) and AT groups (**C**) were investigated with linear models. Abbreviations: dECM = dynamic eigenvector centrality; SD = standard deviation; A- = Amyloid negative; A-T- = Amyloid negative Tau negative; A+T- = Amyloid positive Tau negative; A+T+ = Amyloid positive Tau positive. * = *P* < 0.05, ** = *P* < 0.01, *** = *P* < 0.001.

**Dynamic EC in RSN.** Lower EC dynamics were found in A+ participants in posterior parietal and temporal portions of the DMN (Fig. 5A) and in the dorsal portions of the visual network (Supplementary Fig. 3A). The sensory-motor and frontoparietal networks also showed significant differences between amyloid groups, however, the significant cluster did not exceed 10 voxels and are therefore not discussed. Only a trend to significance was found in the central executive network with A+ having higher dynamic EC (data not shown). A+ participants showed lower variability of EC over time (i.e. SD and range) compared to A- in the DMN (*P* < 0.001***, β = -0.31), and the visual network (*P* < 0.0001***, β = -0.32) (Fig. 5B, Supplementary Fig. 3B). Both A+T- and A+T+ participants showed reduced dynamic ECM SD compared to the A-T- group in the DMN (A+T- = *P* < 0.01**, β = -0.27; A+T+ = *P* < 0.01**, β = -0.45) and in the visual network (A+T- = *P* < 0.001***, β = -0.33; A+T+ = *P* < 0.05*, β = -0.30) (Fig. 5C, Supplementary Fig. 3C).

### EC relationship with cognition

**Figure 6:**
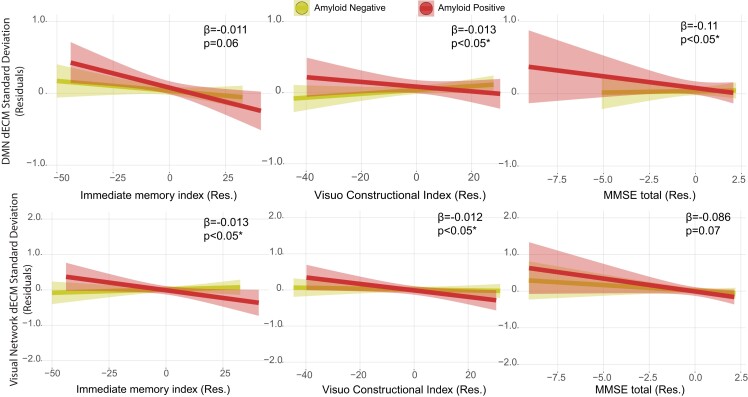
**Relation between cognitive performance and dynamic EC standard deviation in the default mode and visual networks in the amyloid groups.** The RBANS immediate memory index and visuo-constructional index, and the MMSE score showed significant interactions between amyloid status and cognitive scores on networks dynamic centrality temporal variability measures (SD and range). Residuals of the linear models after correcting for age, sex, and education are shown. Abbreviations: dECM = dynamic eigenvector centrality mapping; Res = residuals.

We found significant effects of the interaction between amyloid status and cognitive tests in predicting both SD and range of temporal centrality dynamics in the default mode and dorsal visual network (Fig. 6). In the DMN, while no association of EC variability with cognition was observed in A-, A+ participants showed a significant negative relationship with MMSE (for A+: β = -0.09; *P* < 0.05; for A-: β = 0.01, *P* = 0.66; *P* interaction = *P* < 0.05), and RBANS visuo-constructional index (for A+: β = -0.01, *P* < 0.05; for A-: β = 0.001, *P* = 0.58; *P* interaction = *P* < 0.05); and trend to significant association with RBANS immediate memory index (for A+: β = -0.01; *P* < 0.01; for A-: β = -0.001; *P* = 0.61 ; *P* interaction = *P* = 0.06). Similarly, in the dorsal visual network, only A+ showed negative associations of EC variability with RBANS immediate memory index (for A+: β = -0.01; *P* < 0.01; for A-: β = -0.001; *P* = 0.58 ; *P* interaction = *P* < 0.05); and RBANS visuo-constructional index (for A+: β = -0.01; *P* < 0.01; for A-: β = -0.001; *P* = 0.53 ; *P* interaction = *P* < 0.05). Coefficients in Fig. 6 refer to SDs of dynamic EC, comparable results were observed with range. All model coefficients can be found in the Supplementary Tables 2–6. No significant effect of the interaction of amyloid status and cognitive performance was found on mean static EC within RSNs. In summary, dynamic EC variability in default mode and visual networks were negatively correlated with cognitive performance in the amyloid positive group, but not in the amyloid negative group.

## Discussion

In this study, we showed EC alterations in the pre-dementia stages of AD, using a large sample (*N* = 701) of non-demented participants from the EPAD cohort. Amyloid positivity was associated with static EC reductions in the posterior portion of the brain and EC increases in the frontal and middle cingulate areas. In addition, sliding-window EC variability analysis showed that amyloid deposition is associated with decreased EC dynamics and less variability over time within those areas. Importantly, we found decreased dynamic variability in the default mode and visual networks in A+ participants. Moreover, while static EC in RSNs was unrelated to cognition, dynamic variability measures in these networks did relate to cognitive performance. Our results provide new evidence on the heterogeneous patterns of network changes in the early stages of AD, using centrality measures. We demonstrate the added value of investigating the dynamic aspects of EC, to further characterize the underlying aberrant patterns that might explain static EC alterations in AD signature regions. We propose dynamic EC as a potential early brain biomarker associated with initial amyloid deposition and subtle cognitive alterations.

### Static EC

The observed static EC differences are in line with previous literature in AD.^37^ A reduction of static EC in posterior DMN areas has been previously found in relationship to early amyloid accumulation in cognitively unimpaired individuals.^11,38^ Those studies did not report any increase in centrality in A+ individuals. A possible explanation for our current finding is the choice of region of interests used in the analysis. Focusing on large scale networks may be required to highlight connectivity reductions typical of preclinical stages, while performing modular or voxel-wise analysis uncovers more subtle EC changes in the different network subsystems. Previous works investigating voxel-wise EC have observed similar patterns of centrality alterations, with increased anterior and decreased posterior EC in AD patients compared to controls;^9^ and increased EC in anterior and middle cingulate cortex and decreased EC in the inferior parietal lobule in relationship to CSF p-tau/Aβ1–42 ratio.^12^

Our results on the static EC are also in agreement with previous studies utilizing different FC analysis, such as independent component analysis (ICA) or seed-based FC, and observed regional amyloid-dependent changes in FC. Specifically, increased functional connections in medial prefrontal cortices and decreases in the posteromedial cortex and angular gyrus have been previously observed using ICA approach on rs-fMRI datasets.^39^ Similarly, voxel-wise FC analyses have shown amyloid-related wide-spread hyperconnectivity in non-demented elderly individuals.^40^ In line with those results, we found reduced static EC in posterior parietal and occipital areas and increased EC in frontal areas in relation to amyloid deposition in cognitively unimpaired participants. When looking at static EC across AT groups, we found that most of the observed EC alteration could be replicated in the A+T- group. By contrast, only mild temporal reduction of EC was found in the A+T+ group. While this is in line with the hypothesis of FC being disrupted mainly in relationship to early amyloid deposition, greater impairment would be expected in more advanced stages of the disease. These results might be confounded by the small sample size of the A+T+ group compared to the other two groups and warrants further investigation. Another possible explanation of this result is the proposed existence of nonlinear trajectories of FC alterations throughout the AD spectrum, with early changes that would reverse in more advanced stages.^41^

Our findings conform with the recently proposed *cascading network failure model.*^3^ In this model, an initial amyloid-independent within-network connectivity decline in at-risk individuals in the posterior hubs of the DMN is followed by a compensatory increase in connectivity between posterior and other DMN subsystems to transfer information processing functions. These initial functional alterations would further trigger downstream cellular and molecular events promoting Aβ plaques formation in neocortical layers.

### Dynamic EC

In addition to studying the voxel-wise distribution of static EC in relation to amyloid, we further investigated its dynamic properties, which enable detection of shorter time scale related changes in EC. Specifically, variability in EC would promote an optimal balance between moments of high modularity low efficiency, when different networks are disconnected, and low modularity high efficiency, when those networks interact.^5^ We here investigated variability of dynamics of functional centrality using EC for the first time in the early stages of AD. We found that frontal areas showing higher static EC in A+ participants were characterized by a reduced variability over time of dynamic EC. When looking at canonical RSNs, we further observed that cognitively unimpaired A+ participants had lower EC dynamics and less temporal variability in the visual and default-mode networks, with lowest variation in the absence of cognitive impairment. Dynamic connectivity changes and their relationship with cognition have not been extensively studied in the AD spectrum. In a previous study using different dynamic analysis of FC,^42^ AD patients and patients with Lewy Body Dementia were found to spend more time in more sparsely connected dynamic states of FC compared to controls. Similarly, more recent evidence reported differences in FC dynamic states dwell time between AD patients and control groups and further observed lower dynamic FC variability in Alzheimer patients^43^ and in relationship with transient changes in cognition typical of early stages of neurodegenerative disease.^44^ In line with our results, recent work has also suggested that early amyloid deposition in the brain reduces variations of rs-fMRI BOLD signal^45^ and FC^41^ over time in cognitively unimpaired individuals.

Our findings of reduced EC variability may result from an early amyloid-driven local synaptic loss,^46^ promoting inter-RSN asynchronous activations and intra-network super-synchronous activations,^47^ thus reducing brain functional integration. Indeed, high variability is often observed in functional hubs, possibly related to their participation in different subnetworks. Initial amyloid deposition could therefore cause lower involvement of functional hubs connectivity with different networks, and provoke the prevalence of single dynamic state.^48^ On the other hand, current evidence also suggests patterns of synaptic activation and long-range synchronous activity to influence amyloid production.^49^ The observed patterns might therefore be due to a circular mechanism involving amyloid deposition being both the driver and the result of neuronal activity alterations. Moreover, the observed decreased variability of dynamic EC in A+ participants with good cognitive performance suggests that this reduction of functional integration between RSN in the early stages of AD further promotes cognitive deterioration. However, the lack of longitudinal data and regional information on amyloid burden hampers the interpretation of any directional and regional association between these two phenomena. Taken together, these results propose dynamic connectivity as one of the first functional mechanisms to be impaired by amyloid deposition and stress its promising value as an early predictive biomarker.

### Strengths and limitations

Potential limitations of this study include that EPAD is a research cohort of healthy participants that were specifically selected for their elevated risk for AD, which may not be representative of the general early-phase sporadic AD population. However, the large sample (*N* = 701) and good phenotyping of EPAD are a unique characteristic compared to previous fMRI studies. As opposed to the majority of the previous literature on the topic, a strength of this work is that FC was explored on a voxel-wise centrality base, by using functionalities from the fastECM toolbox.^29^ By mapping the dynamic fastECM measurements within canonical RSNs, we were able to map both the spatial and temporal characteristics of dynamic connectivity. Another limitation is related to the fact some brain regions, such as the ventral part of the cerebellum and the orbitofrontal cortex, were not included in the analysis as not included in the field of view of all centers. As mentioned in the methods, previous works have proposed 100 s to be a safe window-length lower limit for detecting non-spurious fluctuations of FC.^30,31^ However, other studies have shown 300–400 timepoints to be necessary for accurate FC estimation at a single-subject level.^50^ While this work only focused on group inferences, this evidence suggests caution in interpreting dynamic results. Future work will be needed to evaluate the impact of other pathological changes, such as global atrophy, on FC dynamics. This is the first study to describe dynamic graph properties of FC in relationship with early pathological changes of AD.

## Conclusion

We found that investigating dynamic graph properties of FC adds meaningful information to sole investigation of static frame-wise connectivity, and can help in the understanding of early functional pathological events. Our results suggest that initial amyloid deposition affects EC temporal patterns by reducing involvement of functional hubs in different network dynamics, therefore reducing functional integration, and promoting cognitive deterioration.

## Supplementary Material

fcad088_Supplementary_Data

## Abbreviations

AD =: Alzheimer’s disease
CAT =: computational anatomy toolbox
CDR =: clinical dementia rating scale
CEN =: central executive network
CSF =: cerebrospinal fluid
DMN =: default mode network
EC =: eigenvector centrality
EPAD =: European prevention of Alzheimer’s dementia
fastECM =: fast eigenvector centrality mapping
FC =: functional connectivity
fMRI =: functional magnetic resonance imaging
FWHM =: full-width half maximum
GLM =: general linear model
ICA =: independent component analysis
MMSE =: mini-mental state examination
MNI =: Montreal Neurological Institute
RBANS =: Repeatable Battery for the Assessment of Neuropsychological Status
RSN =: resting-state network
SD =: standard deviation
