# Supplementary material for: Eigenvector centrality dynamics are related to Alzheimer’s disease pathological changes in non-demented individuals
Source: Brain Commun. 2023 Mar 28;5(3):fcad088. doi: 10.1093/braincomms/fcad088 (PMC10156145; doi:10.1093/braincomms/fcad088)
Supplement: fcad088_Supplementary_Data [file fcad088_supplementary_data.docx]

Supplementary Materials

#

Supplementary Table 1. Demographic and clinical characteristics of participants in AT groups.

|  | A-T-  n=427 | A-T+  n=42 | A+T-  n=181 | A+T+  n=51 |
| --- | --- | --- | --- | --- |
| Males (%) | 167 (39.1) | 16 (38.1) | 83 (45.9) | 26 (51.0) |
| Age, Mean (SD) | 63.89 (6.78) | 68.95 (6.82) | 63.77 (6.97) | 70.43 (5.24) |
| CDR = 0.5 (%) | 54 (12.7) | 6 (14.3) | 28 (15.5) | 22 (43.1) |
| MMSE, Mean (SD) | 28.89 (1.42) | 28.43 (1.53) | 28.82 (1.66) | 28.02 (1.67) |
| Hippocampal Volume in mm3, Mean (SD) | 2923.59 (486.28) | 2956.94 (300.48) | 2829.67 (469.51) | 2774.14 (560.71) |
| Years of Education, mean (SD) | 14.66 (3.75) | 14.95 (4.24) | 14.76 (3.70) | 13.90 (4.35) |
| RBANS |  |  |  |  |
| Total Scale, Mean (SD) | 109.49 (62.13) | 101.79 (13.83) | 104.41 (12.45) | 94.65 (15.05) |
| Sum of Index, Mean (SD) | 521.91 (53.45) | 506.93 (47.76) | 516.38 (43.46) | 480.59 (57.18) |
| Attention, Mean (SD) | 102.47 (45.93) | 97.64 (16.58) | 99.12 (15.14) | 92.45 (16.71) |
| Delayed Memory, Mean (SD) | 106.08 (44.91) | 101.02 (13.40) | 103.23 (13.53) | 95.33 (18.53) |
| Language, Mean (SD) | 99.68 (9.63) | 99.74 (9.45) | 99.04 (10.62) | 94.88 (11.81) |
| Visuo-Construction, Mean (SD) | 109.45 (15.10) | 106.45 (16.39) | 108.77 (14.93) | 102.73 (14.74) |
| Immediate Memory, Mean (SD) | 106.33 (12.91) | 102.07 (13.31) | 106.22 (12.89) | 95.20 (17.45) |

Maximum CDR in the EPAD cohort is 0.5, therefore the number (and percentage) of CDR = 0.5 is reported. Abbreviations: SD = Standard Deviation; RBANS = The Repeatable Battery for the Assessment of Neuropsychological Status.

#


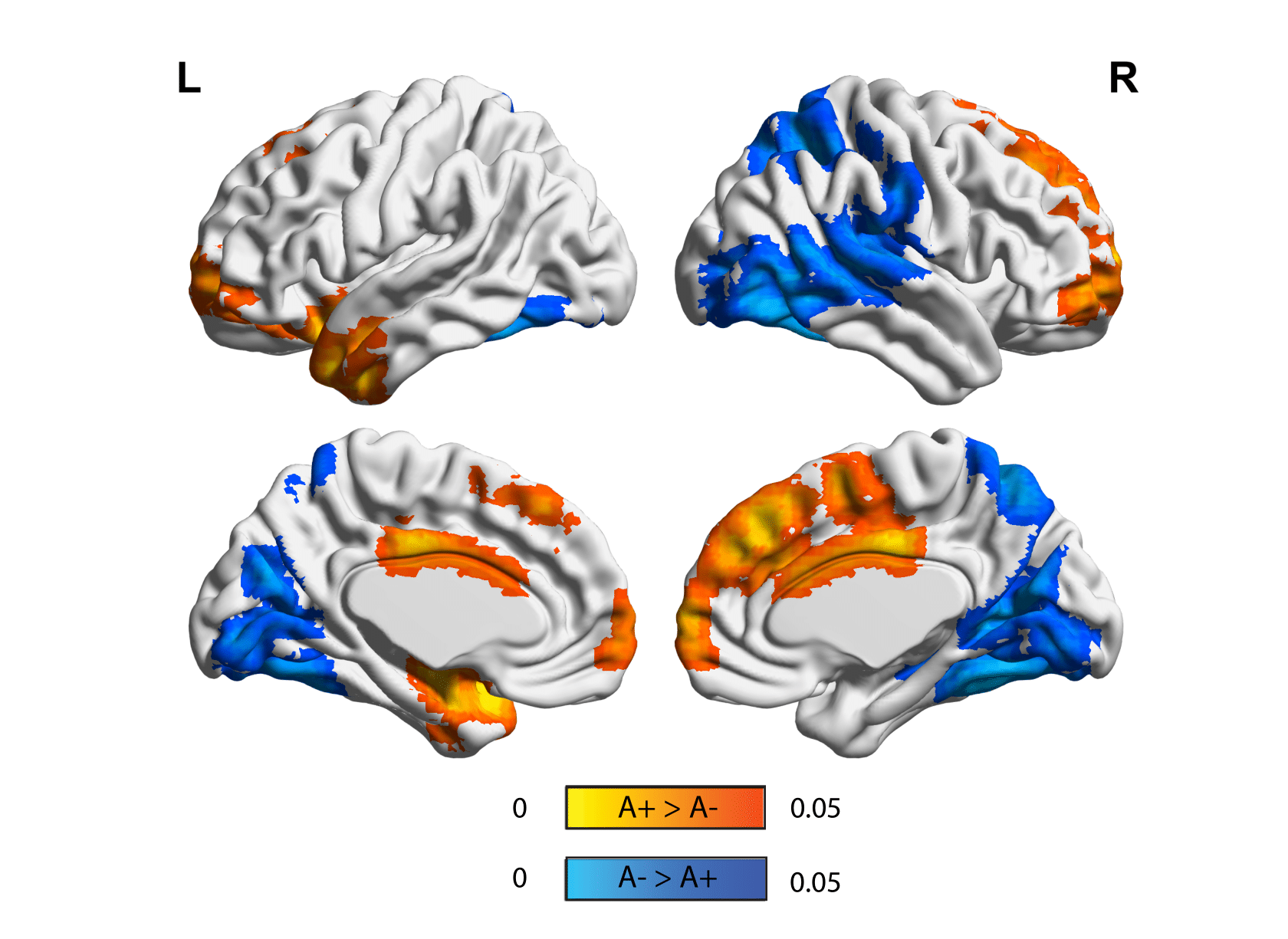


Supplementary Figure 1. Static eigenvector centrality differences in amyloid groups when not excluding suspected non-Alzheimer Pathology participants (A-T+). Surface plots of p-values in statistical significant clusters. Orange shows regions where ECM A+> A- and blue shows regions where ECM A->A+. Upper one is the lateral view, lower row is the medial view, for the left hemisphere (left column) and right hemisphere (right column), respectively.

Supplementary Table 2. Significant ECM cluster information

| Cluster Name | Significant  Contrast | Peak MNI coordinates (x,y,z) | Size (Voxels) | Amyloid Negative  ECM  Mean ± SD | Amyloid Positive  ECM  Mean ± SD | P-value (peak) |
| --- | --- | --- | --- | --- | --- | --- |
| Fronto-temporal | A+ > A- | -38, 6, -36 | 941 | 0.0073 ± 0.0001 | 0.0075 ± 0.0002 | 0.004 |
| Parietal | A- > A+ | 54,-62,60 | 268 | 0.0078 ± 0.0002 | 0.0077 ± 0.0002 | 0.033 |
| Posterior-temporal | A- > A+ | 46,-58,-20 | 1278 | 0.0078 ± 0.0001 | 0.0077 ± 0.0001 | 0.004 |


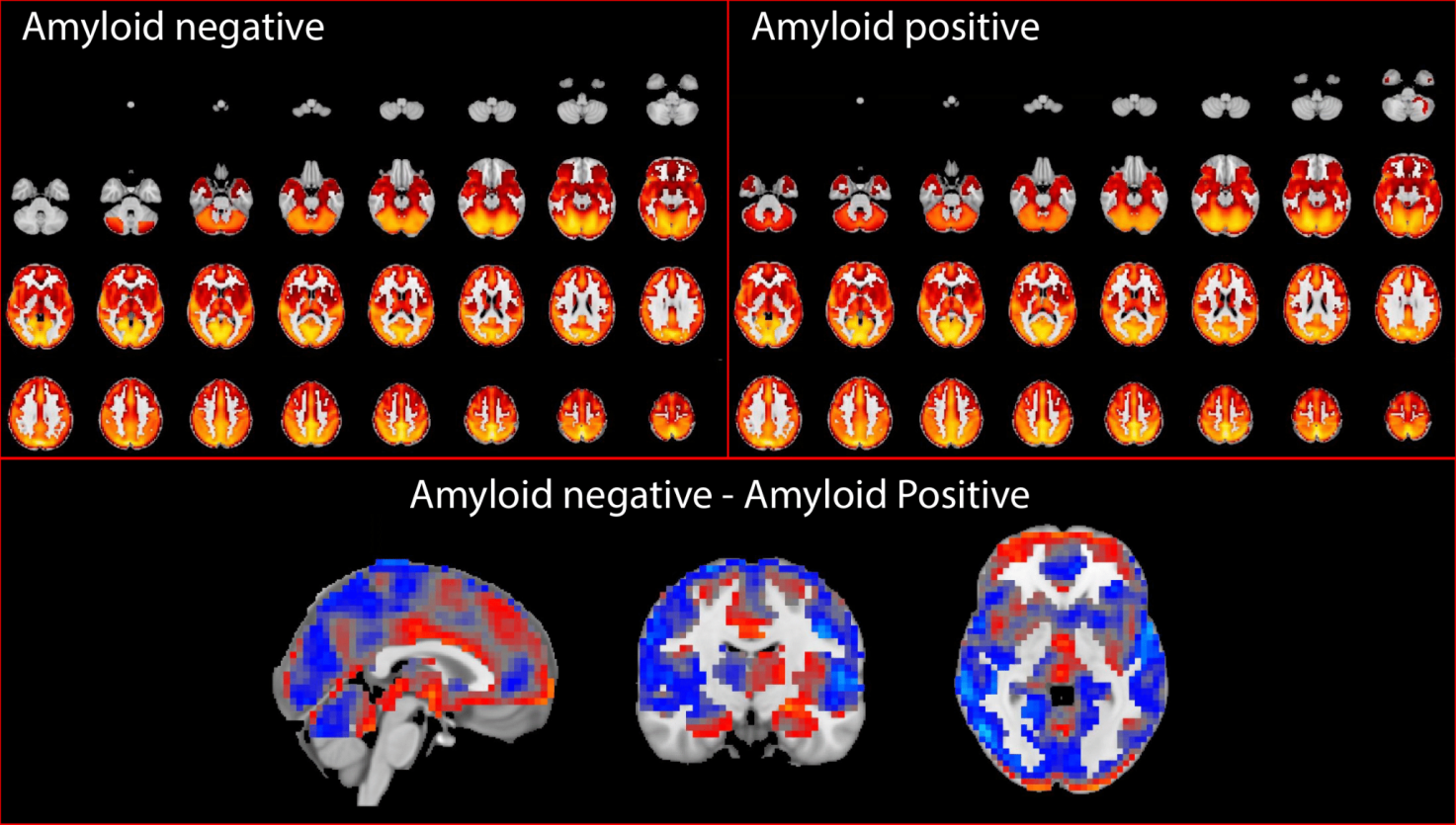


Supplementary Figure 2. Raw voxelwise ECM. Upper-left: mean ECM map in the A- group; Upper-right: Mean ECM map in the A+ group; Bottom: Subtraction of mean ECM in A- and mean ECM in A+.


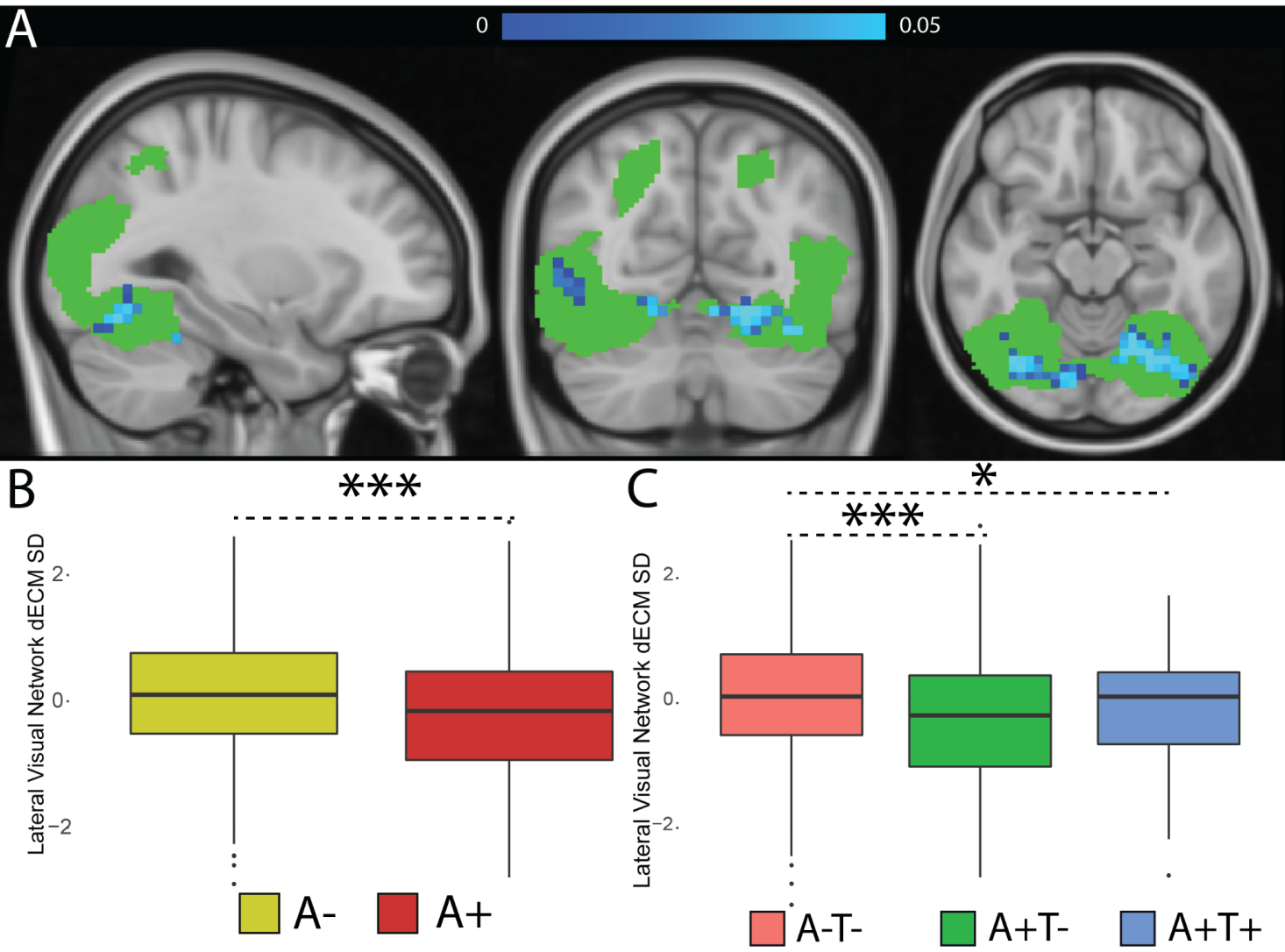


Supplementary Figure 3. Dynamic functional connectivity eigenvector centrality in the dorsal visual network. A) Statistical significant differences showing lower dynamics in amyloid positive participants (in blue). Bottom-row: Differences in dorsal visual network dynamic EC standard deviation (left) between amyloid groups (B) and AT groups (C) participants. Abbreviations: dECM = dynamic eigenvector centrality; SD = standard deviation.

Supplementary Table 3. Effect of the interaction between cognitive performance and Amyloid Status on Visual Network dECM standard deviation

| Predictor | Beta | Standard error | P-value |
| --- | --- | --- | --- |
| Immediate memory * A-status | -0.013 | 0.005 | 0.03 |
| Visuo-constructional Index * A-status | -0.012 | 0.005 | 0.02 |
| MMSE * A-status | -0.090 | 0.05 | 0.07 |

Abbreviations:dECM = dynamic eigenvector centrality mapping; A-status = Amyloid status; MMSE = Mini-Mental State Examination.

Supplementary Table 4. Effect of the interaction between cognitive performance and Amyloid Status on Default Mode Network dECM standard deviation

| Predictor | Beta | Standard error | P-value |
| --- | --- | --- | --- |
| Immediate memory * A-status | -0.011 | 0.006 | 0.06 |
| Visuo-constructional Index * A-status | -0.013 | 0.005 | 0.01 |
| MMSE * A-status | -0.113 | 0.050 | 0.02 |

Abbreviations:dECM = dynamic eigenvector centrality mapping; A-status = Amyloid status; MMSE = Mini-Mental State Examination.

Supplementary Table 5. Effect of the interaction between cognitive performance and Amyloid Status on Visual Network dECM range

| Predictor | Beta | Standard error | P-value |
| --- | --- | --- | --- |
| Immediate memory * A-status | -0.012 | 0.006 | 0.02 |
| Visuo-constructional Index * A-status | -0.012 | 0.005 | 0.02 |
| MMSE * A-status | -0.091 | 0.050 | 0.07 |

Abbreviations: dECM = dynamic eigenvector centrality mapping; A-status = Amyloid status; MMSE = Mini-Mental State Examination*.*

Supplementary Table 6. Effect of the interaction between cognitive performance and Amyloid Status on Default Mode Network dECM range

| Predictor | Beta | Standard error | P-value |
| --- | --- | --- | --- |
| Immediate memory * A-status | -0.011 | 0.006 | 0.06 |
| Visuo-constructional Index * A-status | -0.013 | 0.005 | 0.02 |
| MMSE * A-status | -0.105 | 0.050 | 0.03 |

Abbreviations: dECM = dynamic eigenvector centrality mapping; A-status = Amyloid status; MMSE = Mini-Mental State Examination.
